# ICT infrastructure in firms and online sales

**DOI:** 10.1007/s10660-022-09533-z

**Published:** 2022-02-07

**Authors:** Eva Hagsten

**Affiliations:** University of South-Eastern Norway, Gullbringvegen 36, 3800 Bø, Norway

**Keywords:** Online sales, ICT infrastructure, Broadband, Production function, Cross-country data, Dynamic panel data models

## Abstract

This study investigates whether the underlying information and communication technology (ICT) infrastructure of firms affects the development of online sales, based on a novel micro-aggregated panel dataset encompassing a large group of European countries. The dataset includes continuous measures of online sales activities, as well as of standard production function input variables. Dynamic System GMM estimations show positive and significant associations between the proportion of firms selling online and the ICT infrastructure, measured as the proportion of broadband internet connected employees. The magnitude of the effect is stronger in the group of manufacturing than in the service firms, but in both industries, there is a threshold beyond which the positive effects of the infrastructure diminish. In addition, there is evidence that improvements in the infrastructure lead to a stronger effect on medium-sized and large rather than on small firms.

## Introduction

Early prophecies of the digital economy predicted a rapid global expansion of e-commerce and high valuations of firms engaged in this activity [23, 54, 55]. Indeed, in certain businesses such as book retailers, music retailers and travel agencies, traditional sales channels hardly exist any longer [29]. Despite this, the overall development of online sales is slow, at least in Europe. In 2019, one out of five firms with more than ten employees engage in these activities, although the spread across the 27 European Union member countries goes from ten to 40 per cent (Source: Eurostat Data Browser ISOC_EC_ESELN2). This stands in contrast to a much higher proportion of firms that purchases online and a steady increase in consumer demand for this channel (Sources: ESSLait Micro Moments Database and Eurostat Data Browser ISOC_EC_IBUY,see also Fig. 3 for the dataset used in this analysis).

Besides factors related to the economy in general such as the dot.com bubble, the financial crisis and institutional settings [1, 27, 62], the low level of adoption points to supply side aspects. Literature suggests that typical barriers to the implementation of e-sales systems include trust, risk taking, market power as well as underlying technological infrastructure and distribution channels [2, 13, 26]. More recent research identifies similar obstacles for emerging countries [58]. Several of the aspects mentioned are difficult to measure, although there is a variety of indicators available for the usage of information and communication technology (﻿ICT) in firms, such as for instance the OECD and Eurostat databases.^1^ It is also possible that certain goods and services simply are less suitable for online sales [2].

This study aims to empirically investigate whether the underlying ICT infrastructure in firms helps to explain the spread of online sales (e-sales) in a group of twelve European countries (Austria, Denmark, Finland, France, Germany, Ireland, Italy, the Netherlands, Norway, Sweden, Slovenia and the United Kingdom). The dependent variable represents the proportion of firms with at least one per cent of their sales online; and the independent variable denotes the proportion of employees connected to the internet with a minimum broadband speed, reflecting the underlying ICT infrastructure. This latter variable might harbour effects broader than the technology itself due to unmeasured intangible assets, such as skills of employees [4]. With panel data and continuous variables at hand, the dynamic estimations will be carried out in a framework that mirrors that of a production function model. Particular attention is paid to the degree of connectivity, persistence and to variations across different groups of firms (size-class and industry).

While the effects of ICT investments on firm performance is extensively investigated within production function frameworks both at the level of the firm (see [14] and [28], for reviews of the evidence) and in multi-country industry settings [16, 45, 57], less emphasis is put on how electronic or online sales relates to output. In some cases, there are positive links to productivity, where spillover effects within the specific industry as well as the importance of kind and size of firms is accentuated [15, 21, 36, 40, 47, 60]. Morgan-Thomas and Bridgewater (2004) review the literature on international e-commerce and stress the role of technology and sophisticated use of internet for successful online exports.

Unlike the literature on electronic commerce as driver of output, research on factors affecting the pure business-to-business or business-to-consumer adoption is rich. Several, mainly single-country cross-sectional firm-level studies, often focusing on small and medium-sized firms, highlight the quality of the underlying ICT infrastructure in the decision to sell online [24, 25, 27, 32, 61]. Additional aspects of importance are ICT skills, efficiency, adjustment and transaction costs, interoperability, market reach, organisational readiness, size of firm, security as well as strategic value [11, 12, 20, 30, 31, 37, 41, 48, 52]. There are also the so called *born globals*, which in contrast to other firms do not internationalise incrementally, but compete globally from inception, and are thus highly likely to directly from start depend on digital sales channels [3, 56]. These firms tend to be small and rely on specific technologies, knowledge or innovations [8, 50].

Sila [52] concludes that the empirical literature on e-commerce adoption is often based on small samples of cross-sectional data, and reports contradictory results for the determinants included, such as size of firm. This study contributes to both a deeper and more general understanding of ICT infrastructure as one of the important determinants of the proportion of firms that sell online. Deeper because the underlying Micro Moments Database (MMD) encompasses continuous variables in dimensions not earlier available such as size-class, industry and ICT intensity, and broader due to the large country coverage and longitudinal data.^2^ Data characteristics also mean that the estimation approaches can deviate from the commonly used logistic regression based on cross-sectional data, and take into account persistence over time as well as the magnitude of different explanatory variables (see for instance [9, 33, 46, 49]).

The study proceeds as follows: Next section covers the conceptual background and the empirical approach. A description of the dataset and some stylised facts ensues. Following this there is a presentation of the estimation results and some concluding remarks.

## Conceptual background and empirical approach

**Fig. 1:**
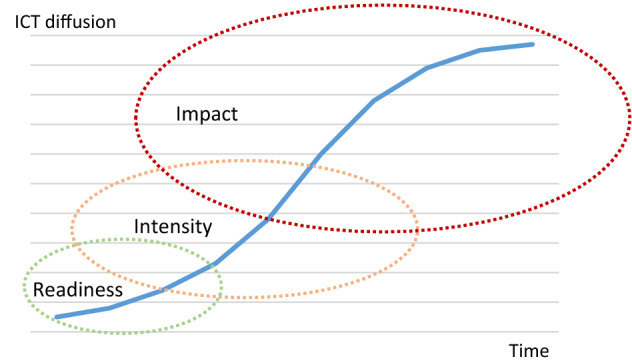
Diffusion of ICT Innovation. *Source*: Own illustration based on OECD [44]

According to theory, diffusion is the process by which an idea or innovation is communicated through different channels among participants in a social system over time [51]. The different stages of this process are recognised as early adopters, early majority, late majority and laggards. OECD [44] employs a refined approach for ICT innovations in firms which identifies three stages: (i) readiness, (ii) intensity and (iii) impact (Fig. 1). Readiness relates to the ability of a firm to adopt an ICT innovation, intensity (or use) measures the proportion of firms that adopt and the extent of use. Impact relates to changes in behaviour, economic structure or performance as a result of use. Firms across countries, industries and size-classes may operate at different stages of diffusion, implying that this could also be reflected in their ability to use advanced applications dependent on an underlying ICT innovation.

Some studies concur with the definition of e-commerce as being an innovation [7, 9, 33, 52, 64] while others rather consider it an advanced computer network application that complements the underlying infrastructure [15, 17, 21, 22, 24].

A joint feature of the research into factors influencing the adoption of e-commerce in firms is the importance of the organisational readiness, often expressed as the underlying ICT infrastructure, technology or skills [18, 19, 33, 35, 38, 39, 46, 52, 53, 61, 63].

In this case, the underlying ICT infrastructure is considered to be the innovation, approximated by the proportion of employees connected to the internet with a minimum broadband speed. This variable encompasses both aspects of the human capital and the underlying technological infrastructure of the firm [4] and is expected to relate significantly and positively to the proportion of firms engaged in online sales. This leads to the formulation of the first hypothesis:

### Hypothesis 1

There is a significant and positive association between the underlying ICT infrastructure and the proportion of firms with online sales.

Another aspect of importance is that adoption may differ among firms of different sizes and sectors [6, 12, 33, 52], something that lies behind the formulation of the second and the third hypotheses:

### Hypothesis 2

The magnitude of the significant and positive association between the underlying ICT infrastructure and the proportion of firms with online sales differs across industry sectors.

### Hypothesis 3

The magnitude of the significant and positive association between the underlying ICT infrastructure and the proportion of firms with online sales differs across size-classes.

Empirical analyses of the probability to adopt e-commerce in firms typically employ probit or logit models based on binary dependent variables [6, 9, 20, 33, 46, 49, 61, 64]. Similar kinds of variables are also often used to reflect the underlying ICT infrastructure in firms, for instance the number of ICT elements in use [6, 33].

ICT impacts, on the other hand, are commonly estimated by use of augmented Cobb Douglas production functions including the inputs labour, capital and materials [14]. This approach allows an interpretation of the extent to which the different inputs relate to output. Several quantifiable variables of importance suggested in the adoption literature coincide with those typically included in a production function. Given the availability of data on these elements, together with measurable independent and dependent ICT variables, the present study employs an approach that mirrors the augmented production function where the proportion of firms selling online (*AESELL*) is the output and (*BROADpct*) is the infrastructure variable. The standard input factors are represented by capital (*K*), labour (*L*) and materials (*M*). Oliveira and Martins [46] highlight the importance of the technological readiness, which may be difficult to uphold without suitable investments, captured by the capital variable. Labour reflects the size of firm and materials the purchases of inputs such as components and services by external experts [9, 20, 32]. Persistence is captured by the inclusion of the dependent variable lagged in time. The infrastructure variable allows time delayed reactions.

The dataset is organised in two alternative ways, allowing dynamic estimations of both two-digit industries (Eq. 1a) as well as size-class and broad industry groups (Eq. 1b):

1a $$AESELL_{ict} = \,\alpha_{ic} +\alpha_{1i} AESELL_{ict-1}+ \alpha_{2i} AESELL_{ict-2}+\beta_{1i} \ln L_{ict} + \beta_{2i} \ln K_{ict} + \beta_{3i} \ln M_{ict} + \beta_{4i} BROADpct_{ict} + \beta_{5i} BROADpct_{ict}^{2} + \lambda_{t} + \varepsilon_{ict}$$

1b $$AESELL_{ijct} = \,\tilde{\alpha }_{ijc} + \tilde{\alpha}_{1i} AESELL_{ijct-1}+\gamma_{1j} \ln L_{ijct} + \gamma_{2j} \ln K_{ijct} + \gamma_{3j} \ln M_{ijct} + \gamma_{4j} BROADpct_{ijct} + \lambda_{t} + \tilde\varepsilon_{ijct}$$

where *i* is 26 two-digit industries, *c* denotes country, *t* reflects year (2003–2010) and $$\lambda_{t}$$ encompasses the time effects. Specification 1b adds the dimension *j* for four size-classes and narrows the industries *i* down to six broad groups. Parameters $$\alpha_{ic}
$$ and $$\stackrel{\sim }{\alpha }_{ijc}$$ hold industry, country and size-classes fixed. The squared term of the broadband variable (*BROADpct*^*2*^) is included to investigate possible thresholds (non-linearities) beyond which the presumptive impact of the ICT infrastructure changes characteristics (see for instance [17].

Based on literature, the direction of causality is assumed to run from the ICT infrastructure to online sales adoption. Nevertheless, endogeneity might occur attributed to unobservable factors affecting both the ICT infrastructure and online sales. Thus, to account for a possible correlation between broadband internet connected employees and the error term, the System GMM panel data estimator [10] is used, where the *BROADpct* is treated as predetermined and the groups refer to size-class, industry and country pairs. This estimator is particularly suitable for datasets with a large number of cross-sectional units and a relatively narrow time frame, as is the case here.

## Data and stylised facts

Data for this analysis stem from the ESSLait Micro Moments Database, available at Eurostat Safe Centre [5].^3^ This database holds linked and micro-aggregated information on firms with ten employees or more originating from the national statistical offices in 14 European countries. Due to laws on disclosure of firm-level data from official sources, there are few opportunities to merge such information into one single international database. A way to partly circumvent this limitation is to work closely with statistical offices that agree to build harmonised dataset that can be micro-aggregated to a level higher than the firm but lower than broad industry-groups.

Information in the Micro Moments Database covers several underlying sources: registers on business, trade and education as well as surveys on production, ICT usage and innovation activities (CIS) in firms for the years 2001–2010. Data are reported for the two-digit industry level, for the EUKLEMS alternative hierarchy (broad industry groups including ICT producers) and in several other dimensions such as size-class, age class, ICT intensity, innovation activity, ownership, affiliation and international experience.^4^ An overview of the industry classification is presented in Table 4, Appendix 1.

In this empirical application, the two-digit industry country panel dataset and data aggregated by both size-class and broad industry groups are employed for twelve countries. This means that the estimation datasets encompass either 26 two-digit industries or six broad industry groups (NACE rev. 1.1: 15a6, 20, 21, 22, 23a4, 25, 26, 27, 28, 29, 30a3, 31, 32, 34, 35 and 36a7 for manufacturing, 50, 51, 52, 55, 60t3, 64, 65t7, 71a4, 72, and 73 for services or Consumer goods, Investment goods, Intermediate goods, Electrical equipment and telecommunication, Financial services and Business services) of which each has four size-classes (10–19, 20–49, 50–249 and 250 + employees). Due to disclosure issues the dataset can only be split in size-classes at the higher aggregation of industries.

The ICT infrastructure of firms is approximated by the proportion of broadband internet connected employees (*BROADpct*), a composite variable reflecting the degree of connectivity among employees within and across firms. This continuous variable is derived from information on the number of employees with broadband internet access of a certain minimum speed and is regarded more sophisticated than categorical and other commonly used basic ICT infrastructure measures relating to the firm (as suggested by for instance [6, 17, 33]) which do not take into account the employee abilities in the same way as the BROADpct variable [4].

Another advantage of the broadband variable is that it does not reach saturation (despite apparent increase) during the period of time studied, which is otherwise not uncommon for fast developing technologies. The capital variable (*K*) is based on either stock or book values, employment (*L*) measures the number of employees and materials (*M*) is defined as the gross value of production minus added value and goods for re-sale. Nominal prices have been deflated by EUKLEMS or WIOD indexes.^5^

**Fig. 2:**
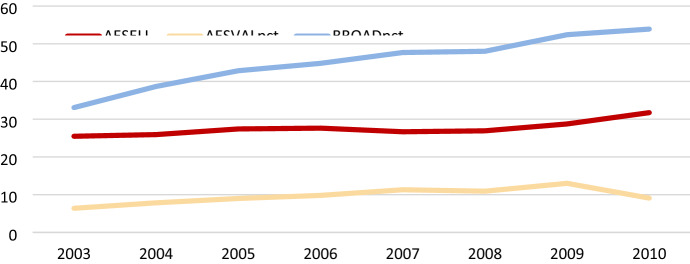
Development of online sales and broadband internet connected employees. *Per cent.* (Note: Data relate to 12 countries: Austria, Denmark, France, Finland, Germany, Ireland, Italy, the Netherlands, Norway, Slovenia, Sweden and the United Kingdom. AESELL illustrates the proportion of firms with online sales, AESVALpct online sales in relation to turnover in firms and BROADpct the underlying ICT infrastructure as the proportion of broadband internet connected employees. *Source*: ESSLait Micro Moments Database)

The proportion of firms selling online increases slightly over the period of time studied while the infrastructure variable is surging much stronger (Fig. 2). Close to a third of the firms engage in online sales, with a slight advantage in manufacturing, while the share of turnover relating to online sales is markedly lower in both manufacturing and service firms, fourteen and ten per cent, respectively in 2010 (Table 1). More than every second employee has a broadband internet connection of a certain speed. In service firms, this human capital related ICT infrastructure occurs even more frequently than in manufacturing.

**Table 1:** Online sales and ICT infrastructure in firms across industries in 2010. *Source*: ESSLait Micro Moments Database and own calculations

Variable, per cent	Manufacturing			Services		
	Mean	Min	Max	Mean	Min	Max
Proportion of firms selling online (AESELL)	31	8	42	29	9	44
Proportion of online sales (AESVALpct)	14	3	35	10	3	28
Proportion of broadband connected employees (BROADpct)	42	23	66	57	35	76

Average across 12 countries: Austria, Denmark, France, Finland, Germany, Ireland, Italy, the Netherlands, Norway, Slovenia, Sweden and the United Kingdom. The industry classification refers to NACE 1.1 manufacturing 15t37 and to services 50t74

**Fig. 3:**
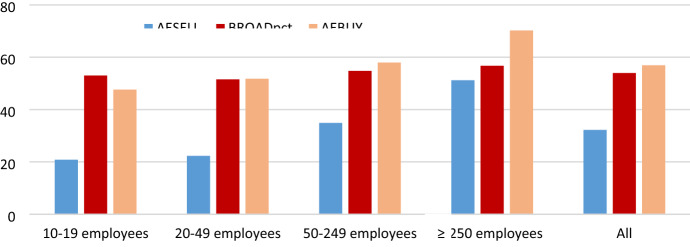
Online sales, purchases and ICT infrastructure by size-class, in 2010 *Per cent. *Note: Data relate to 12 countries: Austria, Denmark, France, Finland, Germany, Ireland, Italy, the Netherlands, Norway, Slovenia, Sweden and the United Kingdom. Variables AESELL, AEBUY and BROADpct mean proportion of firms engaged in online sales or purchases and proportion of broadband internet connected employees. *Source*: ESSLait Micro Moments Database

As suggested in literature, the adoption of e-sales applications may vary not only among industries but also across size-classes (Fig. 3). In this case, large firms engage in sales more than twice as often as small firms. No such systematic difference can be found for the ICT infrastructure variable, which is generally on a higher level of intensity than online sales. All firms are also much more active with online purchases (*AEBUY*) than sales, possibly indicating that this is a far less complex and costly issue to deal with.

## Results and discussion

The dynamic system GMM estimations disclose that there is a significant and positive association between the underlying ICT infrastructure, approximated by the proportion of broadband internet connected employees, and the proportion of firms selling online in the group of countries studied (Table 2, Specification (i)). This result imply that Hypothesis 1 cannot be rejected. The same is valid for Hypothesis 2, since the magnitude of the coefficients is larger for the manufacturing firms with less experience of this infrastructure than for the service firms.

**Table 2:** Impact of ICT infrastructure on proportion of firms selling online, by industry. *System GMM estimations*. *Source*: ESSLait Micro Moments Database and own calculations

A. Manufacturing firms 2003–2010	(i)		(ii)	
Dependent variable: Proportion of firms selling online	Coeff	t-stat	Coeff	t-stat
Online sales (t-1)	0.62^***^	10.29	0.62***	8.74
Online sales (t-2)	0.18^***^	3.18	0.17***	2.80
ln employment (t)	0.01	0.59	0.04*	1.74
ln capital (t)	− 0.01	− 1.42	− 0.01**	− 2.20
ln material inputs in constant prices (t)	− 0.01	− 0.50	− 0.03	− 1.57
Broadband internet connected employees in % (t)	0.69^***^	4.75		
Broadband internet connected employees in % squared (t)	− 0.66^***^	− 4.14		
Broadband internet connected employees in % (t-1)			0.41***	3.83
Broadband internet connected employees in % squared (t-1)			− 0.34***	− 2.82
Time effects	Yes		Yes	
Constant	0.05	0.51	0.15*	1.80
Number of observations	1191		1191	
Number of groups (country-industry pairs)	168		168	
Number of instruments	150		150	
Hansen test of over-identifying restrictions (*p*-value)	0.21		0.14	
Arellano-Bond test for AR(1) (*p*-value)	0.00		0.00	
Arellano-Bond test for AR(2) (*p*-value)	0.37		0.45	
**B. Service firms 2003–2010**	**(i)**		**(ii)**	
Dependent variable: Proportion of firms selling online	Coeff	t-stat	Coeff	t-stat
Online sales (t-1)	0.40***	3.60	0.36***	3.18
Online sales (t-2)	0.15	1.39	0.16	1.48
ln employment (t)	0.00	0.13	0.00	− 0.13
ln capital (t)	0.00	0.41	0.00	− 0.31
ln material inputs in constant prices (t)	0.01	0.47	0.03	1.13
Broadband internet connected employees in % (t)	0.48**	2.39		
Broadband internet connected employees in % squared (t)	− 0.43**	− 2.36		
Broadband internet connected employees in % (t-1)			0.44*	1.98
Broadband internet connected employees in % squared (t-1)			− 0.36*	− 1.81
Time effects	Yes		Yes	
Constant	− 0.25	− 1.53	− 0.28**	− 2.09
Number of observations	558		558	
Number of groups (country-industry pairs)	83		83	
Number of instruments	85		85	
Hansen test of over-identifying restrictions (*p*-value)	0.39		0.40	
Arellano-Bond test for AR(1) (*p*-value)	0.12		0.08	
Arellano-Bond test for AR(2) (*p*-value)	0.97		0.78	

Asterisks ^***, **^, and ^*^ denote significance at the 1, 5, and 10 per cent levels, respectively. The two-step GMM estimator based on Windmeijer correction for small samples and robust standard errors is used. Variable broadband internet connected employees is treated as predetermined. In addition, the Hansen J-test supports the validity of the instruments at the one per cent significance level in all cases (*p*-value) and the AR(2) test shows that there is no second-order serial correlation

Not only the contemporaneous level of broadband connected employees but also its lagged term relates positively to the proportion of firms selling online (Table 2, Specification (ii)), although the former is stronger. In addition, the estimations reveal that online sales is more persistent, or path-dependent in the manufacturing than in the service industry, implying that the proportion of firms selling online in earlier years influences the present adoption. This could also mean that the service firms are more flexible and quicker to adjust.

**Fig. 4:**
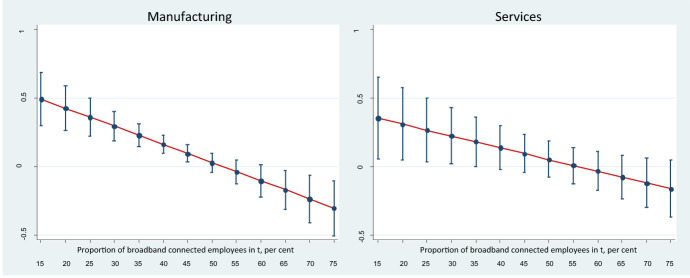
Short run marginal effect of broadband internet connected employees on the proportion of firms selling online. Note: The graphs illustrate the marginal effects including the 95 per cent confidence intervals at different proportion of the infrastructure variable BROADpct. From the point where the confidence interval crosses the horizontal zero line, the significant effect disappears. *Source*: ESSLait Micro Moments Database and own calculations

Given the strong growth of the infrastructure variable, a possible significant and positive effect might change characteristics after a certain threshold. In this case, the estimates of the squared broadband term relate significantly negatively to the proportion of firms selling online, somewhat stronger in manufacturing than in service firms, indicating a non-linear relationship where the effect of the infrastructure variable is decreasing with higher usage. In order to give an idea of the magnitude of the relationship, the marginal effect is calculated, based on the proportion of broadband connected employees. This uncovers that, with a proportion of broadband connected employees of 25 per cent, an increase by ten percentage points gives a marginal effect of three percentage points for service and four percentage points for manufacturing firms (Fig. 4).^6^ The calculation also reveals that beyond a threshold of 50 per cent in manufacturing firms and 45 per cent in service firms, the short run ICT infrastructure boost to the proportion of firms engaged in online sales disappears. This aligns with the law of diminishing marginal utility, implying that when a certain level of the infrastructure is reached this is no longer a factor of importance for the choice to sell online.

Further, the results uncover that the standard production factors are not significantly different from zero or show the wrong sign. Several explanations may lie behind this result. One of them could relate to the aggregation level of results. Another possibility is that negative effects of labour are attributed to a reverse job-saving. ICT is sometimes expected to save jobs, especially in the short run (see for instance [59]). A decrease in the proportion of firms that sell online could follow from investments that require more physical staff, for instance in the super warehouses that compete with online sellers, as suggested by Hortaçsu and Syverson [34]. Alternatively, strategies to raise investments, use additional intermediates or hire new employees without paying attention to their specific quality or skills, do not necessarily stimulate an increase of firms that sell online.

Turning to the estimations by size-class, additional details are exposed. The relationship between online sales activities and the ICT infrastructure does vary across size-classes as stated in Hypothesis 3 (Table 3). It is generally more pronounced for the groups of large and medium-sized firms. This is somewhat unexpected since these firms are already more intensive users of online sales applications and there is no major difference in the level of broadband connected employees among size-classes. The path dependency is also stronger for the largest firms. Possibly, other factors such as kind of products or services, supply chain management [37], advantages of scale, sales volumes (units) and client categories (business to businesses, consumers or governments) may be of importance for the choice of sales channels, although information on all this is not available in the dataset at hand.

**Table 3:** Impact of ICT infrastructure on firms selling online, by size-class. *System GMM estimations. * *Source*: ESSLait Micro Moments Database and own calculations

	(i)		(ii)		(iii)		(iv)	
Dependent variable:	10–19 employees		20–49 employees		50–249 employees		250 + employees	
Proportion of firms selling online	Coeff	t-stat	Coeff	t-stat	Coeff	t-stat	Coeff	t-stat
Online sales (t-1)	0.40***	5.28	0.36***	4.54	0.45***	5.90	0.67***	11.13
ln employment (t)	− 0.01	− 0.40	− 0.07***	− 3.02	− 0.08**	− 2.14	0.01	0.77
ln capital (t)	0.00	− 0.47	− 0.01	− 1.53	− 0.01	− 1.42	0.00	0.06
ln material inputs in constant prices (t)	0.01	0.52	0.05**	2.39	0.04	1.61	− 0.01	− 0.51
*Broadband internet connected*								
employees in % (t)	0.08***	2.68	0.07*	1.86	0.13**	2.07	0.15**	2.10
Time effects	Yes		Yes		Yes		Yes	
Constant	0.07	0.57	0.16	0.70	0.58***	2.70	0.05	0.17
Number of observations	406		406		406		406	
Number of groups (country-industry pairs)	56		56		56		56	
Number of instruments	76		76		76		76	
Hansen test of over-identifying								
restrictions (*p*-value)	0.93		0.93		0.87		0.87	
Arellano-Bond test for AR(1) (*p*-value)	0.00		0.00		0.00		0.00	
Arellano-Bond test for AR(2) *p*-value)	0.23		0.07		0.31		0.04	

Asterisks ***, ** and * denote significance at the 1, 5 and 10 per cent levels. The estimations by size-class exclude Ireland and Slovenia. The two-step GMM estimator with Windmeijer correction for small samples and robust standard errors is used. Variable broadband internet connected employees is treated as predetermined. In addition, the Hansen J-test supports the validity of the instruments at the one per cent significance level in all cases (p-value) and the AR(2) test shows that there is no second-order serial correlation except in the case of large firms

The results verify earlier firm-level research in that the underlying infrastructure is of importance for the adoption and diffusion of e-commerce and that it might vary across firm size [6, 12, 33]. However, the evidence in this study is both more specific and general: Specific following the panel data set with clearly defined continuous ICT variables in several dimensions that allows comparisons of magnitudes, and general due to data on the representative firm by industry and size-class for twelve European countries.

Several robustness checks are undertaken. To investigate whether the model is mis-specified, the estimations are also carried out with the more traditional production variable *turnover of online sales*, despite its quality shortcomings. These estimations (available upon request) reveal a pattern similar to that of the main results, but with smaller magnitudes and weaker significances. Employment, capital and materials turn out equally unimportant. An implication of these results is that during the period of time studied, the proportion of firms selling online could be interpreted as an approximation of the extent to which firms sell online, since both variables develop similarly but on different levels.

Given the possibility of self-selection into online sales by reasons of efficiency or competitiveness [32, 42], for instance, estimations are also performed with the ICT infrastructure instrumented by the level of labour productivity in groups of firms. Although labour productivity would be directly related to the extent of sales online, the main dependent variable reflecting the proportion of firms selling online is not expected to exhibit the same association. The average labour productivity in constant prices over time is Euro 59,000 for the manufacturing firms and Euro 76,000 for the services firms (Source: Micro Moments Database). The high amount for services reflects an overrepresentation of business services in the dataset. The estimations reveal that the infrastructure variable is still significant for both services and manufacturing firms (results available upon request).

Despite the use of System GMM for the estimations, the possibility of reverse causality cannot be fully neglected. Because of this, the main specifications for manufacturing and services firms are estimated with reverse order for the two ICT variables, ceteris paribus. This renders significant results for the manufacturing but not for the services firms (Table 6, Appendix 1). Since the variables are scaled differently, a one standard deviation change is calculated to investigate which effects dominate. The standard deviations are 0.4 and 0.3 for BROADpct and AESELL, respectively. This implies that the impact on online sales is 40 per cent and that the reverse effect is 4 per cent. Thus, the overall causality goes from infrastructure to online sales.

**Fig. 5:**
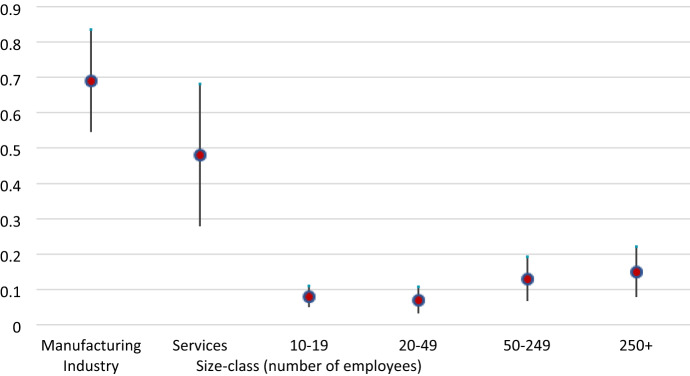
Variable BROADpct 95 per cent confidence interval across estimations. Note: The graph illustrates the point estimates (red dot) with 95 per cent confidence intervals of the variable BROADpct for the separate regressions on different industries and size-classes (Tables 2 and 3 in the main body of the text). *Source*: ESSLait Micro Moments Database and own calculations

To verify that the comparisons of estimates across different sub-groups of firms are statistically valid, the 95 per cent confidence intervals for the BROADpct variable are plotted (Figure 5, Appendix 1). These intervals overlap to a certain extent, implying that the sub-groups are not significantly different from each other and thus the comparison of the magnitude of point estimates are valid.

## Concluding remarks

By use of a novel micro-aggregated dataset encompassing a large group of European countries, and by departing from the standard approach to use binary variables for adoption of ICT, this study investigates the importance of the underlying ICT infrastructure of firms for the extent to which they engage in online sales activities. Online sales activities are with few exceptions (travel agencies and book retailers, for instance), still not widespread among firms. However, large firms more routinely use this sales channel. Dynamic System GMM estimations based on a specification that mirrors a production function show positive and significant relationships between the proportion of firms selling online and their underlying ICT infrastructures, measured as the proportion of broadband internet connected employees. The effect is stronger for manufacturing firms and more persistent over time than for service firms. However, there are indications that the possible boost diminishes after the infrastructure reaches a certain threshold.

When the size-classes are estimated separately the results reveal that the group of large firms, already more experienced in online sales activities, benefit the most from an improved ICT infrastructure. This could relate to advantages of scale, supply chain management or to the kind of production. The standard production factors capital, labour and materials do not show significant and positive links to the proportion of firms selling online. Possibly, this means that specific rather than general skills and quality of the inputs are needed to stimulate firms to sell online. Instead, the results point to the importance of both human capital and technology for the online sales activities. Resistance to online sales may also indicate that there is a certain amount of goods and services that is less suitable for this channel such as fresh produce and typical items that require physical inspection before the purchase.

Although novel variables, broad country and industry coverage, time series spanning over several years as well as an alternative estimation approach allow both more specific and general conclusions, the study has some limitations. It does not give insights into the exact behaviour of firms or the level of online sales that is adopted. Due to data quality issues, the proportion of firms engaged in online sales is used as the main dependent variable, while the more traditional, turnover based one only occurs in the robustness check. This check leads to similar patterns of the results, although with smaller magnitudes and weaker significances. Because of this and due to the fact that these two online sales variables exhibit almost identical trends during shorter periods of time without changes in definitions and coverage, the underlying infrastructure is expected to associate also with the extent of sales online. Just like in firm-level analyses, the data themselves affect the econometric approach. In this case, the two-digit NACE rev. 1.1 classification is more thorough for manufacturing firms, making the panel data approach in lags and levels extra sensitive in the case of the service firms.

There are several avenues for future research: one is to include more countries, update the dataset and prolong the period of time studied. This would allow an investigation into how the outbreak of the Covid-19 pandemic in 2020 affects the proportion of firms that engages in online sales. Another possibility is to investigate alternative variables or instruments for the ICT infrastructure that could be used. Further, the complementarity between the ICT infrastructure and the quality of human capital would deserve a closer look as would an analysis where the firms have been grouped by their position in the value chain or their competitive status.

## Appendix 1

See Fig. 5 and Tables

**Table 4:** Industry definitions (NACE 1.1)

TOT	Total economy
15t37	Manufacturing
15a6	Food, beverages and tobacco
17t9	Clothing
20	Wood and of wood and cork
21a2	Pulp, paper, publishing
21	Pulp, paper and paper
22	Publishing and printing
23t25	Refining, chemicals, and rubber
23a4	Refining and chemicals
25	Rubber and plastics
26	Other non-metallic mineral
27a8	Metals and machinery
27	Basic metals
28	Fabricated metal
29t33	Machinery and equipment
29	Machinery, nec
30t3	Equipment
30a3	Office, accounting and computing machinery; sc. eqpt
31	Electrical equipment
32	Electronic equipment
34a5	Motor vehicles and transport equipment
34	Motor vehicles, trailers and semi-trailers
35	Transport equipment
36a7	Misc manufacturing
40a1	Electricity, gas and water supply
45	Construction
50t74	Market services
50t5	Trade, hotels, restaurants
50t2	Trade, hotels, restaurants
50	Sale, maintenance and repair of motor vehicles and motorcycles; retail sale of fuel
51	Wholesale trade and commission trade, except of motor vehicles and motorcycles
52	Retail trade, except of motor vehicles and motorcycles; repair of household goods
55	Hotels and restaurants
60t4	Transport and communications
60t3	Transport
64	Post and telecommunications
65t7	Banking
70t4	Real estate and bus services
70	Real estate activities
71t4	Business services
71a4	Renting of machinery and equipment; oth. bus. svc
72	Computer and related activities
73	Research and development
75t99	Social services
75	Public admin and defence; compulsory social security
80	Education
85	Health and social work
90t3	Personal services
90t3x	Personal services excl. media
921t2	Media activities

4,

**Table 5:** EUKLEMS alternative industry definitions. *Source*: www.euklems.net

ALT	Description
Elecom	Electrical machinery, post and communication services
MexElec	Total manufacturing, excluding electrical
ConsG	Consumer manufacturing
IntmdG	Intermediate manufacturing
InvesG	Investment goods, excluding hightech
OtherG	Other production
MServ	Market services, excluding post and telecommunications
Distr	Distribution
FinBu	Finance and business, except real estate
Pers	Personal services
NonMar	Non-market services

5,

**Table 6:** Impact of firms selling online on ICT infrastructure, by industry. *System GMM estimations. * *Source*: ESSLait Micro Moments Database and own calculations

	Manufacturing firms				Service firms			
Dependent variable:	(i)		(ii)		(iii)		(iv)	
Broadband internet connected employees (t)	Coeff	t-stat	Coeff	t-stat	Coeff	t-stat	Coeff	t-stat
Broadband internet connected employees (t-1)	0.389***	8.97	0.402****	9.31	0.294***	3.36	− 0.023	− 0.14
Broadband internet connected employees (t-2)	0.379***	7.53	0.405***	8.59	0.421***	8.13	0.332***	3.01
ln employment (t)	− 0.007	− 0.52	0.003	0.24	0.042*	1.69	0.065	1.19
ln capital (t)	− 0.009**	− 2.55	− 0.006	− 1.58	− 0.019***	− 2.92	0.017	1.14
ln material inputs in constant prices (t)	0.004	0.31	− 0.002	− 0.21	− 0.034	− 1.42	− 0.103*	− 1.89
Proportion of firms selling online (t)	0.124***	3.80	0.222***	2.71	− 0.033	− 0.37	0.525	1.51
Proportion of firms selling online squared (t)			− 0.176	− 1.50			− 0.941**	− 2.20
Time effects	Yes		Yes		Yes		Yes	
Constant	0.224***	2.95	0.155**	2.02	0.571***	3.28	0.981***	3.21
Number of observations	1193		1193		567		567	
Number of groups	168		168		83		83	
Number of instruments	166		163		88		89	
Hansen test	0.313		0.25		0.375		0.411	
Arrelano-Bond for AR(1) p-value	0		0		0		0	
Arrelano-Bond for AR(2) p-value	0.071		0.049		0.27		0.23	
Short -run coefficient proportion of firms selling online (t) at sample means			0.116***	4.81			− 0.040	− 0.30

Asterisks ^***, **^, and ^*^ denote significance at the 1, 5, and 10 per cent levels, respectively. The two-step GMM estimator based on the Windmeijer correction for small samples and robust standard errors is used. Variable proportion of firms selling online and its squared term are treated as predetermined. In addition, the Hansen J-test supports the validity of the instruments at the one per cent significance level in all cases (p-value) and the AR(2) test shows that there is no second-order serial correlation

6.

## Footnotes

1 https://www.oecd.org/digital/broadband/broadband-statistics/ and https://ec.europa.eu/eurostat/web/digital-economy-and-society/data/database.

2 Bartelsman, Hagsten and Polder [5].

3 http://ec.europa.eu/eurostat/web/microdata/micro-moments-dataset.

4 EUKLEMS, initiated within the EU Sixth Framework Programme, provides comparable growth and productivity accounts for most European countries, see www.euklems.net.

5 See www.euklems.net and www.wiod.org.

6 Due to the high degree of persistence of online sales and the time period of seven years available, focus is put on the short-run effects.
